# Vocal folds irregular mucosal changes: a multimodal evaluation for diagnosis and genetic risk stratification

**DOI:** 10.1007/s00405-026-10249-1

**Published:** 2026-05-11

**Authors:** Essam Eldin Mohamed Aref, Amira Hafez AbdelAal, Rania Mohamed Ewida, Reham Abdel Wakil Ibrahim

**Affiliations:** 1 Phoniatric Unit, ENT Department, Faculty of Medicine, Assiut University, 71516 Assiut, Egypt; 2 Molecular Biology Researches and studies institute, Assiut University, Assiut, Egypt

**Keywords:** Vocal folds cancer, Irregular mucosal changes, Video-laryngo-stroboscopic, Head and neck genetic, SOX2

## Abstract

**Purpose:**

Irregular vocal fold mucosal changes pose diagnostic challenges and may range from benign to malignant. Emerging research highlights Sex-determining Region Y-box 2 (SOX2) gene amplification as a potential early marker in head and neck tumorigenesis. This work aimed to evaluate vocal fold irregular mucosal lesions for the early detection of high-risk lesions (high-grade dysplasia, carcinoma in situ, and squamous cell carcinoma (SCC)) and to explored the potential role of SOX2 gene in early laryngeal carcinogenesis.

**Methods:**

This is a prospective observational cross-sectional study with a follow-up up to 2 years; 40 male patients with vocal fold irregularities were examined by laryngo-stroboscopy, then classified into two groups based on histopathological diagnosis: (A) Low-risk group (benign lesions, low-grade dysplasia), (B) High-risk group (high-grade dysplasia, carcinoma insitu and SCC. Real-time polymerase chain reaction PCR was performed to detect SOX2 gene expression.

**Results:**

Significant differences were observed in vocal fold stroboscopic features (mucosal wave and amplitude), and SOX2 gene expression across lesion groups, with higher SOX2 expression noted in the high-risk group (*P* < 0.05). A strong positive correlation was observed between histopathological severity and each of stroboscopic impairment in mucosal wave and amplitude, and Log SOX2 gene expression. The regression analysis showed a significant independent predictive effect of SOX2 gene expression on the progression to high-risk lesions.

**Conclusions:**

This study integrated established diagnostic standards, laryngostroboscopy for clinical assessment, and histopathology as the gold standard for grading, while the analysis of SOX2 gene expression showed a promising predictive molecular marker for tumorigenesis.

## Introduction

Leukoplakia, erythroplakia, and keratosis are some of the mucosal surface alterations that can be seen in patients with vocal fold abnormal mucosal changes, which are typically accompanied by other tissue irregularities [1]. These noticeable alterations in the mucosa can be caused by a variety of disorders, from invasive squamous cell carcinoma (SCC) to benign keratosis with or without atypia, and they can also be signs of premalignancy or malignancy. The lack of consensus on surgical criteria and protocols makes this a frequent problem in phoniatric clinics, making diagnosis and treatment more difficult [2].

One of the most important and common ways to evaluate the vocal folds’ vibratory functions and morphology is via laryngostroboscopy. It is possible to detect infiltrative processes in the vocal folds early using stroboscopic examination [3]. Premalignant lesions and their precise delineation can not always be adequately assessed with a laryngoscope examination [4].

Gugatschka et al. [3] revealed that by combining cytology with videostroboscopy, a sensitivity of over 97% may be achieved, compared to 74% when cytology is used alone, allowing for the early diagnosis of infiltrative processes in the vocal folds. Zhang et al. [5]suggested a system for morphologically categorizing three subtypes of vocal fold (VF) leukoplakia. Features like as color, thickness, and texture were assessed. In comparison to type I, which is characterized by flat and smooth leukoplakia, type III, which is bulge and rough with irregular and nonhomogeneous leukoplakia that extends above the mucosal surface, was found to be more frequently linked to cancerization in 29.3% of cases and severe dysplasia in 21.5% of cases. Anna Rzepakowska et al. [6] provided a method for assessing the leukoplakia’s morphological properties, which included the lesion’s size, color, texture, thickness, symmetry, and vibratory function. The characters were given a score between zero and one, with zero indicating benign lesions and one indicating cancer.

While histopathology is the gold standard and stroboscopy is a standard clinical tool, there is a clinical need for objective molecular markers that could predict the risk of malignant progression in lesions that are histologically similar or where biopsy is not immediately pursued.

Advances at the basic science level have led to a deeper understanding of the molecular processes that drive laryngeal cancer in its earliest stages. Chromosome 3 at 3q26 contains the Sex-determining Region Y-box 2 (SOX2) gene, which is often overexpressed and amplified in several malignancies, one of which is head and neck squamous cell carcinoma (HNSCC). The broad demonstration of SOX2’s tumor-promoting activities and participation in tumor development makes it an exciting therapeutic target for cancer therapy. Inadequate information is known about SOX2’s involvement in carcinogenesis or its potential function in malignant transformation at this time [7].

Granda-Diaz et al. examined the SOX2 gene in precancerous lesions using real-time polymerase chain reaction (PCR) for the first time and discovered that during the first phases of laryngeal carcinogenesis, gene amplification and SOX2 protein expression are common occurrences [7].

This work aimed to evaluate vocal fold irregular mucosal lesions for the early detection of suspicious precancerous lesions, which would aid in better management and outcome. Additionally, it aimed to explore the potential role of SOX2 gene expression within different histopathological grades and laryngostroboscopic findings of precancerous vocal fold irregular mucosal changes. SOX2, as a key stem cell factor, was investigated for its potential role in this early laryngeal carcinogenesis pathway.

## Patients and methods

This exploratory prospective observational cross-sectional study with comparison was conducted between March 2020 and August 2023, after approval from the Committee, Faculty of Medicine (Code: 17200388), and registered on clinicaltrials.gov. The study was conducted on 40 male patients aged 47–69 years old, complaining of dysphonia with unilateral or bilateral localized and diffuse vocal fold irregularities on laryngeal examination, leukoplakia patches or erythroplakia on one or both vocal folds, free of any head, neck malignancy, chest, and neurological disease, with no previous history of radiotherapy.

In line with the principles outlined in the Declaration of Helsinki, the ethical protocols were rigorously followed. The patient gave their written informed consent. Candidates were not eligible for inclusion if they had any of the following conditions: allergies, vocal fold immobility, cysts or polyps in the vocal folds, nodules, Reinke’s edema, or contact granulomas. Those patients who were not good candidates for general anesthesia, as well as those for whom the possibility of an airway blockage or abnormally fast development required immediate surgical intervention.

Based on the results of the histopathological analysis, the patients were categorized into two groups. Group(A) Patients with low-risk lesions (benign and low-grade dysplasia, including mild and moderate dysplasia), Group(B) those with high-risk lesions (high-grade vocal fold dysplasia, including severe dysplasia up to carcinoma in situ, and glottic SCC).

## Patient’s interview (history taking)

Data was collected from recruited patients, including age, onset, course, duration of symptoms, Tobacco use, alcohol abuse, occupational exposures to specific chemicals, vocal demand, voice abuse, education level, and marital status.

### Voice assessment

It included auditory perceptual assessment of voice (APA) and laryngeal examination. APA: was performed by two expert phoniatricians using modified grade, roughness, breathiness, asthenia, and strain (GRBAS) scale (kotby et al.) [8]: overall grade (G): Normal (Grade 0), Mild dysphonia (Grade I), Moderate dysphonia (Grade II) severe Dysphonia (grade III) or aphonic (grade IV), character (quality): strained, leaky, breathy or irregular.

The laryngeal examination included both external laryngeal examination and an elementary visual impression of the glottis.

## Visual augmentation

A portable 90° rigid telescope (Xion CH01-D) or an orolaryngoscopic 90° telescope (R Wolf 445057 - Storz/Hopkins) was used during the procedure. Using a Telepack X LED TP100 storz.c for a videostroboscopy analysis. In videostroboscopic analysis the following parameters were reported; *amplitude*: (normal, decreased, absent), *mucosal wave*: (normal, limited, absent), *symmetry*: (symmetrical or asymmetrical in phase and in amplitude), *phase closure*: (complete or incomplete), *glottic gap* (no glottic gap or glottic gap more than 1 mm), and non-vibrating segment.

## Surgical procedure

The patients were initially counselled on the concept, advantages, and possible complications of the procedure. Informed consents, including the possibility of urgent tracheostomy if airway obstruction occurred, were obtained. The patients fasted for a minimum of six hours. The surgery was performed with general anaesthesia under strict aseptic conditions. Laryngeal exposure was performed in the usual way using a suspension laryngoscope and an operating microscope (Carl Zeiss OPMI 1-Fc, Germany) with a 400 mm objective lens. The biopsies were obtained by punch biopsy forceps. Excisional biopsy with safety margin was obtained from localized and small glottic lesions. Each biopsy was divided into two divisions. One division was fixed in 10% formalin for histopathological examination. The other division was preserved in a sterile container and was frozen under − 80^°^C till the time of PCR examination.

## Histopathological examination

Formalin was used to preserve all specimens, and then they were embedded in paraffin. Deparaffinization was carried out in xylol to remove the paraffin from serial five-micron-thick sections cut from the paraffin-embedded tissue blocks. Hydration was carried out using descending alcohols, with absolute, 95%, 90%, 80%, and 70% concentrations (each for five to ten minutes). After a two-minute rinse in distilled water, the slices were stained with hematoxylin for two to twenty minutes. Following a brief staining process with eosin, the sections were rinsed under running water from the faucet. Following a thorough washing in distilled water, the specimens were subjected to a series of escalating alcohol dehydration procedures lasting two to five minutes each in 70%, 80%, 95%, and 100% alcohols. The specimens were then cleared in xylol. The sections were mounted in dibutyl phthalate polystyrene xylene (DPX) and covered with a cover slip. The H&E stained sections were examined and commented after evaluation of the epithelial thickening (any increase in the prickle cell or basal cell layer), keratinization of the epithelium, alteration of cellular differentiation and stratification; atypical cytological features (Pleomorphism, increased nuclear-cytoplasmic ratio, mitotic figure and nuclear crowding), the degree of atypia and the basement membrane (intact or infiltrated).

## SOX2 expression by quantitative real-time PCR (q-PCR)

The total ribonucleic acid (RNA) was extracted by ABT RNA mini extraction kit (Applied biotechnology) according to the kit protocol, and the concentration of RNA was adjusted to the same level by using a spectrophotometer (Gene Quant 1300).

**Complementary DNA (cDNA) synthesis** was carried out using ABT H-minus MMLV cDNA synthesis kit (Applied Biotechnology, USA), where cDNA synthesis was carried out in 20 µL reaction volumes, and the samples were adjusted to the same concentration using a spectrophotometer (Gene Quant 1300).

### Real-time PCR

The real time PCR was used to determine the gene expression of sox gene using the following primers: For SOX2 target gene sequence of primer CATCACCCACAGCAAATGAC (sense) and CAAAAGCTCCTACCGTACCACT (antisense). Β-actin (reference house-keeping gene) CTTAGTTGGGTTAACCCTTTCTTG (sense) and CTGTCACCTTCAGGGTTCCAGTTT (antisense).

The reaction carried out 20 µl which 10 µl of 2× SYBR Green PCR Master Mix (HERA SYBR^®^ Green qPCR Kit), 1 µl of each primer (10 picmol), 5 µl of nuclease free water and 3 µl of cDNA running the experiment on Stratagene^®^ (Mx3000P^®^ qPCR, USA) real time PCR system for normalization of the expression used (Gapdh, B-actin) as an internal control. Thermal profile conditions, initial denaturation step 95 °C for 5 min, then 40 cycles of 45 s at 95 °C, 45 s at (annealing temp for two primers), respectively, and 45 s at 72 °C. The relative fold gene expression of the samples was calculated using 2^-ΔΔCT^ method. The ΔΔCT (cycle threshold) method represents the difference between ΔCT of precancerous and cancerous – ΔCT of non-neoplastic mucosa, with ΔCT being the average CT of the target gene (SOX2) minus the average CT for the reference gene (β-actin), and the patients were categorized into positive SOX2 expression > 1 and negative expression < 1.

## Control cases

The benign cases by histopathology were used as internal control for calculation of ΔΔCT (fold gene expression).

**Regular follow-up**: every three months for the low-grade dysplasia and every month for the high-grade dysplasia for one to two years. Patients underwent a diagnostic protocol for voice assessment. Laryngovideostroboscopic findings were documented. During the follow-up period, patients were carefully examined for any signs of recurrence or growth. If no changes were observed, a biopsy was not performed. At any sign of progression, recurrence, or stroboscopic fixation, lesions were biopsied.

### Statistical analysis

Statistical analysis was performed using SPSS (version 27.0, IBM Corp., Armonk, New York). Quantitative data were presented as mean ± standard deviation, and categorical data as frequency (number, percentage). Group comparisons were made using the Chi-square test (categorical variables) and the Kruskal-Wallis test (continuous, non-normally distributed variables). Correlations were assessed with Spearman’s correlation, and the prediction of malignant transformation by SOX2 gene expression was identified using binary logistic regression. A two-sided P-value < 0.05 was considered statistically significant. Logarithm transformation for fold gene expression values was done then tested statistically. This allowed stabilization for variance for large values that had high variance, and reducing the influence of extreme outliers that could otherwise drive statistical significance.

## Results

**Fig. 1:**
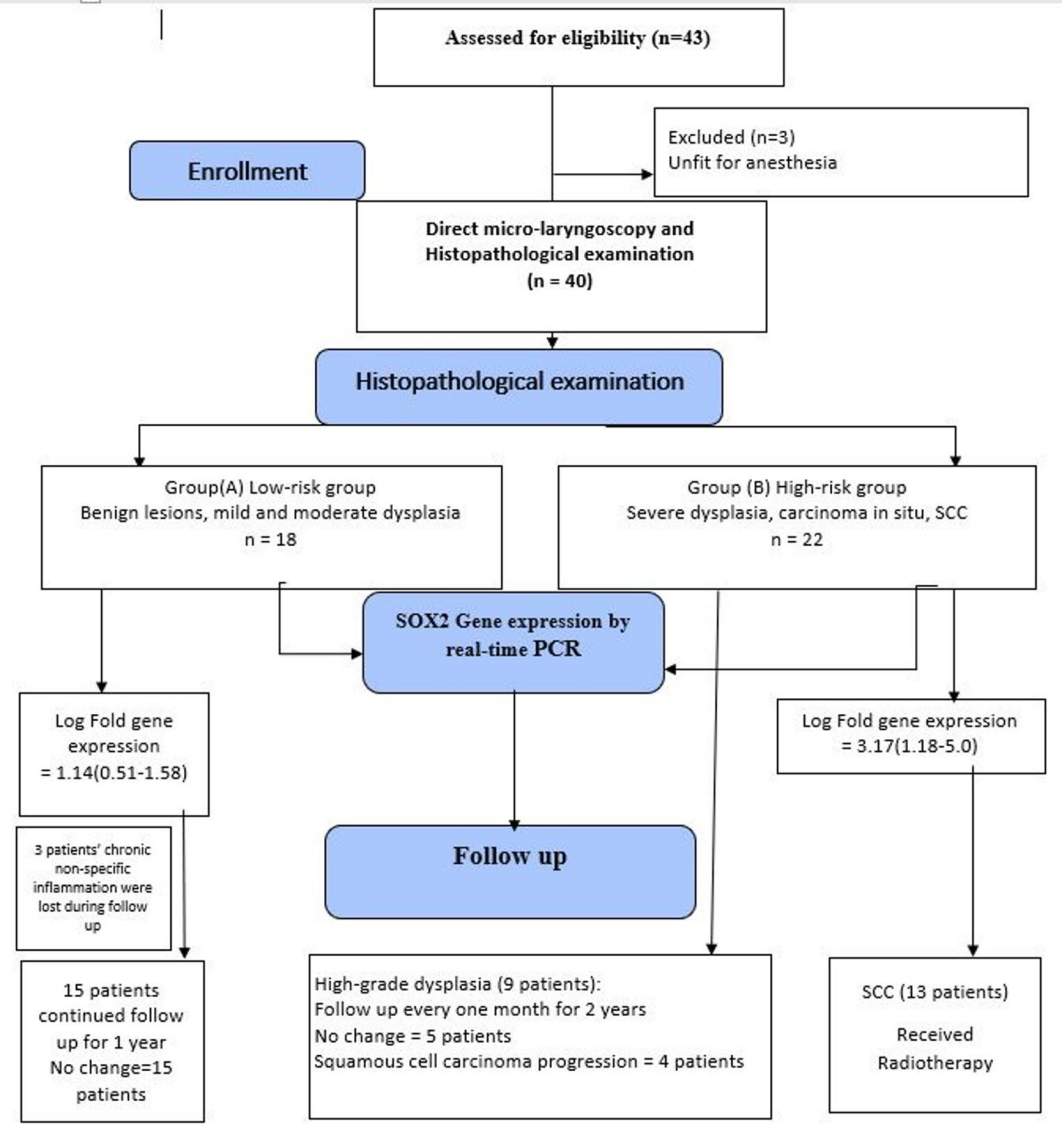
Illustrates the CONSORT flow chart

Out of 43 male participants with vocal folds irregular mucosal changes that were examined with video-laryngo-stroboscopy, 3 patients were excluded because they were unfit for surgery. Forty patients were allocated according to histopathological results into 2 groups: group (A) [low-risk group], including benign lesions and low-grade dysplasia (mild and moderate dysplasia), and group (B) [high-risk group], including high-grade dysplasia(severe dysplasia up to carcinoma in situ), and SCC. All patients were examined by real-time PCR for SOX2 gene expression. Precancerous lesions were followed up for 2 years for the detection of any progression to malignancy (Fig. 1).

**Regarding demographic data of the patients**, the two groups did not differ significantly, *P* > 0.05 Table 1.

**Table 1:** Demographic data of the studied groups

		Histopathological results		*P*-value
		Group (A) Low-risk lesions	Group (B) High-risk lesions	
		*n* = 18	*n* = 22	
Age (Years)		58.39 ± 8.29	61.73 ± 8.49	0.219
Smoking index		325(90–900)	780(500–1000)	0.145
Tense temperament		18(100%)	22(100%)	-
phonasthenic manifestations		18(100%)	22(100%)	-
History of voice abuse		18(100%)	22(100%)	-
Frequency of upper respiratory tract infection	Frequent	3(16.67%)	4(18.18%)	1.000
	Not frequent	15(83.33%)	18(81.82%)	
Hyperacidity and reflux (GERD)		5(27.78%)	4(18.18%)	0.705

The data were presented as mean and Standard deviation for parametric data or were presented as median and quartiles [median (Q1-Q3)] for non-parametric continuous data and for categorical data were presented as number (percentage)*: Statistically significant at *P* ≤ 0.05

GERD: Gastroesophageal Reflux Disease

Chi-square and Kruskal-Wallis tests were used

### Classification by histopathological examination

Patients were distributed as follows: Group (A) low-risk group, including benign lesions and low-grade dysplasia (mild and moderate dysplasia): 18 patients (45%) of the total cases. Group (B) high-risk group: High-grade dysplasia (severe dysplasia and carcinoma in situ) and squamous cell carcinoma consisted of 22 patients (55%).

### Auditory perceptual assessment

Overall grading of dysphonia and character of dysphonia were insignificantly different between the two groups. *P* > 0.05 Table 2.

**Table 2:** Auditory perceptual assessment of the groups studied

		Histopathological results		P-value
		Group (A) Low-risk lesions	Group (B) High-risk lesions	
		*n* = 18	*n* = 22	
Overall grading of dysphonia	Grade 0 (normal)	0(0%)	0(0%)	0.745
	Grade I	2(11.11%)	3(13.64%)	
	Grade II	10(55.56%)	9(40.91%)	
	Grade III	6(33.33%)	10(45.45%)	
	Grade IV (aphonic)	0(0%)	0(0%)	
Character of dysphonia	Strained	18(100%)	19(86.4%)	0.926
	leaky	6(33.3%)	8(36.4%)	
	Breathy	0(0%)	0(0%)	
	Irregular	10(55.6%)	12(54.5%)	

Data is presented as frequency (percentage)

*: Statistically significant at *P* ≤ 0.05

Chi-square test was used

### Vocal folds clinical examination

There was no statistically significant difference between the two groups Table 3.

**Table 3:** Vocal fold examination of the studied groups

			Histopathological results		*P*-value
			Group (A) Low-risk lesions	Group (B) High-risk lesions	
			*n* = 18	*n* = 22	
Vocal fold diffuse irregularities	Homogeneity of lesion color in the vocal fold	Homogenous	7(50%)	2(20%)	0.210
		Heterogenous	7(50%)	8(80%)	
	Lesion texture	Regular	4(36.36%)	1(16.67%)	0.600
		Irregular	7(63.64%)	5(83.33%)	
	Lesion Size	Small	6(42.86%)	1(10%)	0.172
		Moderate	8(57.14%)	9(90%)	
	Lesion hyperemia	Absent	6(42.86%)	1(10%)	0.172
		Present	8(57.14%)	9(90%)	
	Lesion thickness	Thin	4(28.57%)	0(0%)	0.114
		Thick	10(71.43%)	10(100%)	
	Lesion transparency	Shiny	1(7.14%)	0(0%)	1.000
		Opaque	13(92.86%)	10(100%)	
Vocal fold localized irregularities	Size	Small	0(0%)	1(100%)	-
	Surface	Smooth	1(14.29%)	1(8.33%)	1.000
		Irregular	6(85.71%)	11(91.67%)	
	Color	White	5(71.43%)	4(33.33%)	0.170
		Red	2(28.57%)	8(66.67%)	
	Girth	Normal	0(0%)	1(8.33%)	0.066
		Mildly increased	6(85.71%)	3(25%)	
		Moderately increased	1(14.29%)	6(50%)	
		Markedly increased	0(0%)	2(16.67%)	

Data are presented in frequency (percentage)

*: Statistically significant at *P* ≤ 0.05

Chi-square and Kruskal-Wallis tests were used

**Fig. 2:**
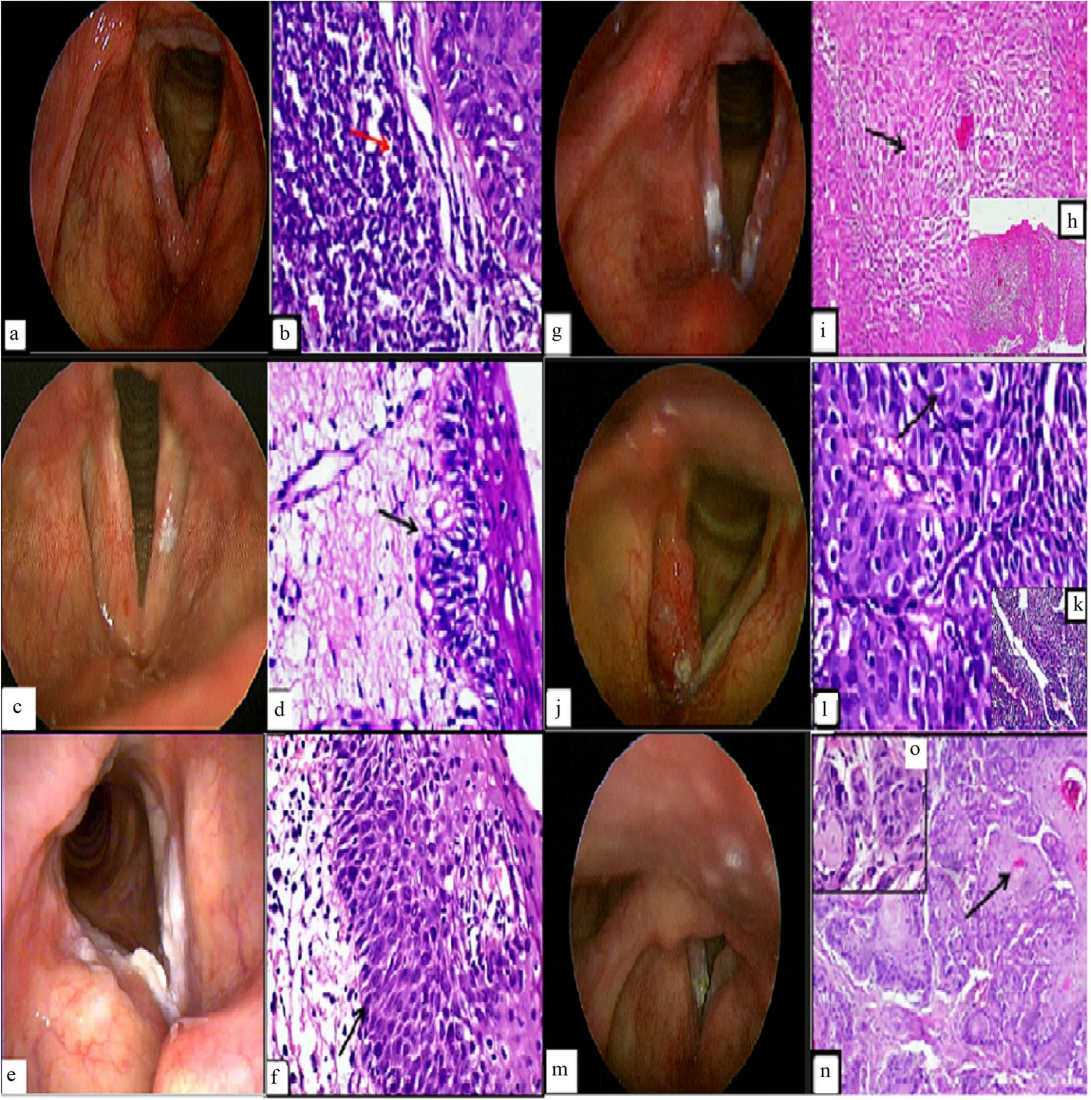
Video-laryngo-stroboscopic examination and histopathological results of various vocal folds lesions: (**a** &**b**) A case of bilateral vocal folds irregularities and hyperemia, histopathological findings suggest chronic non-specific inflammation with a heavy lymphocytic infiltrate in the subepithelial tissue (arrow). (**c** &**d**) A case of diffuse thickening and hyperemia of the covering mucosa with a prominent mucosal leukoplakic patch on the superior surface of the middle third of the left vocal fold. Histopathological examination shows mild dysplasia in the form of a mildly disordered basal layer with mild nuclear atypia (arrow) in the lower third of the epithelium. (**e** &**f**) A case of irregular keratotic lesions of both vocal folds, histopathological examination shows moderate dysplasia in the form of moderate nuclear atypia (arrow) with occasional prominent nucleoli in the lower two-thirds of the epithelium. (**g**, **h** &**i**) A case of diffuse thickening, hyperemia, irregularity, and scattered leukoplakic patches of both vocal folds. Histopathological examination shows severe dysplasia with loss of maturation greater than two-thirds of the epithelium. Higher power of severe dysplasia shows nuclear atypia with hyperchromatic pleomorphic nuclei involving greater than two-thirds of the epithelium (arrow). (**j**, **k** &**l**) A case of huge irregularities occupying almost the whole length of the right vocal fold extending to the anterior commissure with smooth intact mucosal covering and a small, rounded leukoplakic patch in the anterior third of the right vocal fold. Histopathological examination reveals carcinoma in situ that shows full-thickness nuclear abnormalities without stromal invasion. Higher power of carcinoma in situ shows full-thickness nuclear atypia with highly pleomorphic hyperchromatic enlarged nuclei (arrow) and prominent nucleoli. (**m**, **n** &**o**) A case of keratotic irregularity that arises from the middle third of the right vocal fold. Histopathological examination shows squamous cell carcinoma invasion of the subepithelial tissue by variable nests of malignant squamous cells (inset) with areas of keratin pearl formation (arrow)

The following Fig. 2 shows examples of video-laryngo-stroboscopic examination of vocal folds lesions and their corresponding histopathological results.

### Laryngo-stroboscopic assessment

Significant differences were observed in stroboscopic characteristics among the two groups concerning mucosal wave, amplitude of vibration, symmetry in phase, and Non-vibrating segment (Stroboscopic fixation). (*P* = 0.001, 0.02, 0.005, and 0.001, respectively) Table 4.

**Table 4:** Comparison between laryngo-stroboscopic assessment of the studied groups

		Histopathological results		*P*-value
		Group (A) Low-risk lesions	Group (B) High-risk lesions	
		*n* = 18	*n* = 22	
Mucosal wave of the vocal fold	Normal	11(61.11%)	1(4.55%)	< 0.001**
	Limited	5(27.78%)	12(54.55%)	
	Absent	2(11.11%)	9(40.91%)	
Amplitude of vibration of VF	Normal	8(44.44%)	2(9.09%)	0.002**
	Decreased	10(55.56%)	12(54.55%)	
	Absent	0(0%)	8(36.36%)	
Symmetry in phase	Symmetrical	12(66.67%)	5(22.73%)	0.005**
	Asymmetrical	6(33.33%)	17(77.27%)	
Symmetry in amplitude	Symmetrical	11(61.11%)	7(31.82%)	0.064
	Asymmetrical	7(38.89%)	15(68.18%)	
Glottic closure	Complete	4(22.22%)	3(13.64%)	0.680
	Incomplete	14(77.78%)	19(86.36%)	
Glottic gap	No glottic gap	5(27.78%)	3(13.64%)	0.430
	Glottic gap>1 mm	13(72.22%)	19(86.36%)	
Non-vibrating segment (Stroboscopic fixation)		1(5.55%)	16(72.73%)	< 0.001**

Data are presented in frequency (%)

*: Statistically significant at *P* ≤ 0.05

**: Highly significant *P* < 0.01

VF: Vocal fold

Chi-square test was used

**Fig. 3:**
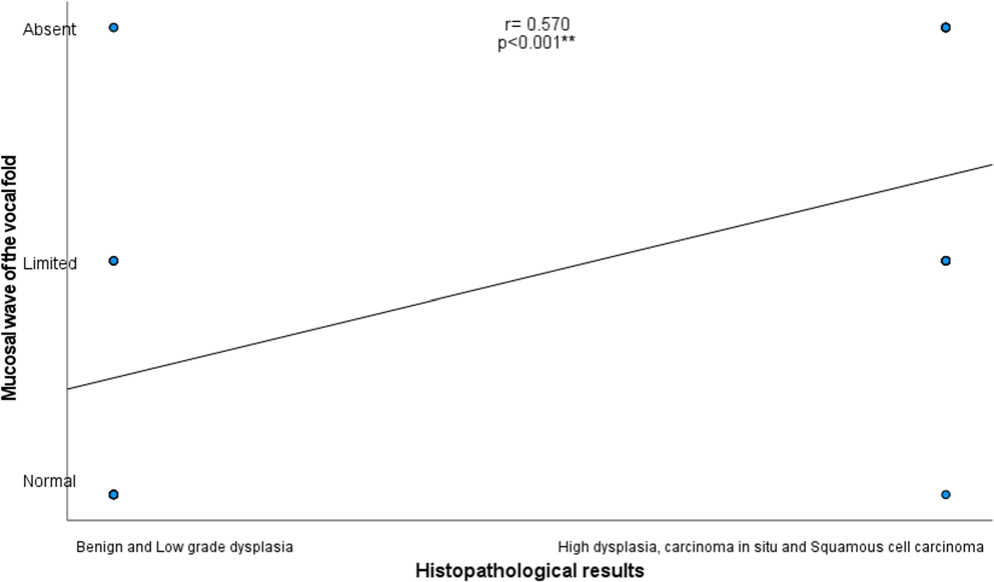
Correlation between VF mucosal wave and histopathological groups

**Fig. 4:**
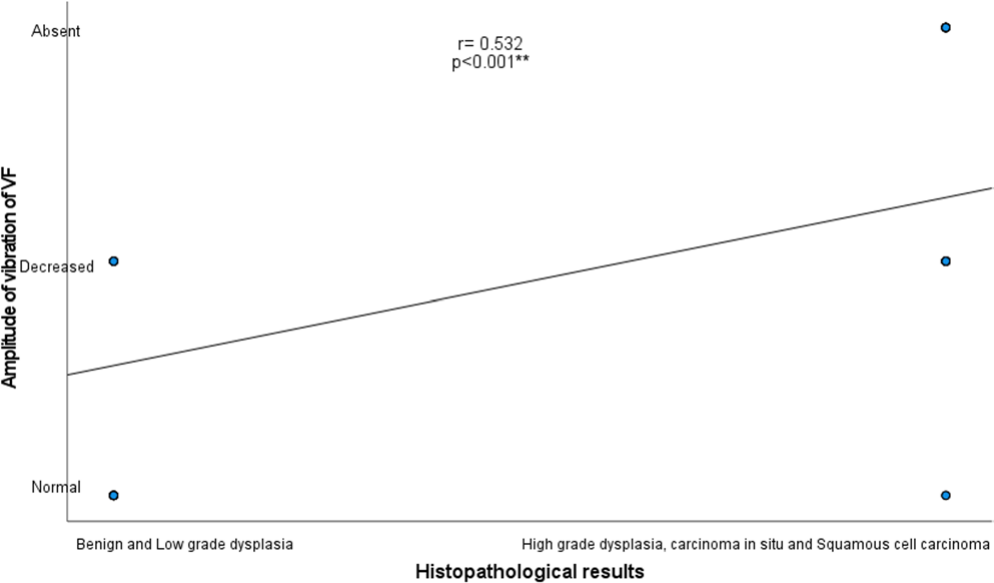
Correlation between the amplitude of vibration of VF and histopathological groups

In (Figs. 3 and 4), there was a positive correlation between histopathological examination and laryngo-stroboscopic assessment parameters (mucosal wave and amplitude of vibration of vocal folds), (*r* = 0.57 and 0.53, respectively, *P* < 0.001 in both, indicating that impairment of vocal fold pliability occurs with higher lesion severity.

### SOX2 gene expression

**Fig. 5:**
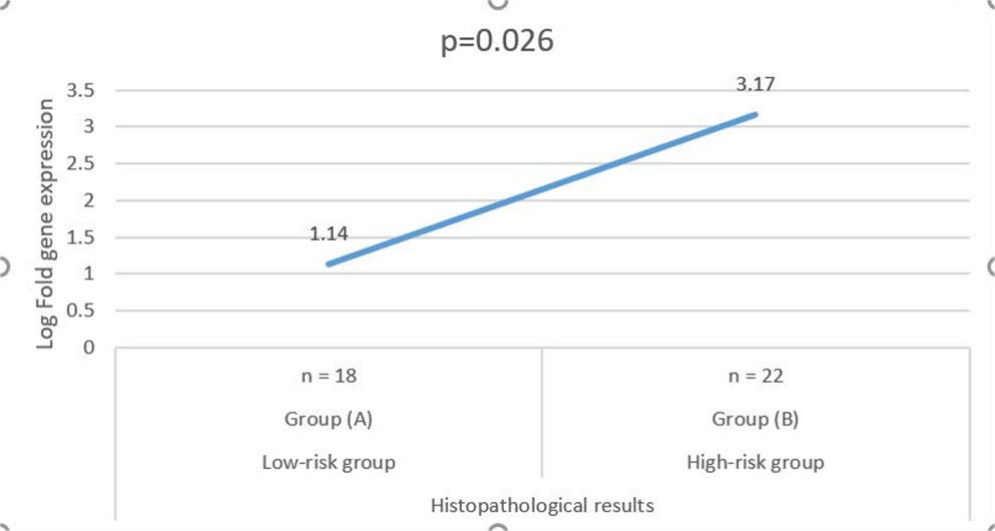
SOX2 gene expression of the studied groups

Log SOX2 gene expression was significantly different among the two groups (*P* = 0.026). While the benign and low-grade dysplasia groups showed low SOX2 gene expression, consistent with minimal expression in early lesions, the high-grade dysplasia and SCC group demonstrated a significant increase in gene expression, implicating higher SOX2 gene expression in advanced premalignancy Fig. 5 and Table 5.

**Table 5:** SOX2 gene expression of the studied groups

	Histopathological results		*P*-value
	Group (A) Low-risk lesions	Group (B) High-risk lesions	
	*n* = 18	*n* = 22	
Log Fold gene expression	1.14(0.51–1.58)	3.17(1.18-5.0)	0.026*

Data are presented as median (interquartile range Q1-Q3)

*: significant *P* < 0.05

The Mann-Whitney U test was used

Histopathological analysis showed a significant positive correlation with Log SOX2 gene expression (p-value < 0.001, *r* = 0.514) Table 6.

**Table 6:** Correlation between histopathological results and SOX2 gene expression

	Histopathological examination	
	*r*	*p*
Log Fold gene expression	0.514	< 0.001

r = correlation coefficient (0-0.3 = weak correlation, 0.4–0.6 = moderate correlation, 0.7-1 = strong correlation)

The Spearman correlation test was used

### Predictors of histopathological risk status (Table 7)

**Table 7:** SOX2 gene rule in histopathological progression into invading squamous cell carcinoma

	B	Bias	Std. Error	*P*-value Sign.(2-tailed)	95.0% CI Lower-Upper Bound
Age	0.015	-0.136	2.968	**0.750**	-1.73–0.233
job/environment	-1.630	-6.885	103.565	**0.056**	-21.812–0.075
smoking index	0.000	0.006	0.097	**0.899**	-0.002–0.006
Log Gene Expression value	1.233	1.896	18.861	0.006**	-15.088–20.581

Dependent Variable: Histopathological results

CI: Confidence Interval

Linear logistic regression test

* significant p-value

** highly significant *p*-value

Four independent variables: Age, job/environment, smoking index, and Log Gene Expression value were tested as predictors for histopathological staging (either low-risk group or high-risk group). Regarding Log SOX2 gene expression, The log-transformed gene expression was associated with a significant prediction to the high-risk histopathological outcome (p-value = 0.006). While age (*p* = 0.750), job/environment (*p* = 0.056), and smoking index (*p* = 0.899) were not statistically significant predictors .

## Discussion

The clinical management of vocal fold irregular mucosal changes, such as leukoplakia, erythroplakia, or diffuse thickening, remains a significant challenge in laryngology. These visible alterations encompass a broad range of pathological conditions. They may represent benign hyperkeratosis, varying grades of dysplasia, or invasive SCC. This heterogeneity complicates diagnosis and treatment, as lesions that seemed similar could have different outcomes (unpredictable behavior of dysplastic lesions). Their potential for recurrence and progression to malignancy requires balancing complete lesion removal with the preservation of vocal function [9].

Building on the standard techniques of **laryngo-stroboscopy** and **histopathological** examination, this study explored **SOX2 gene expression** as a predictive molecular marker for tumorigenesis and malignant progression.

Laryngo-stroboscopy serves as a critical diagnostic method for assessing vocal fold vibratory function. This technique enables the early identification of infiltrative changes within the vocal folds. Key stroboscopic parameters include the mucosal wave and the amplitude of vibration. A preserved mucosal wave with propagating vibration amplitude typically signifies a non-invasive leukoplakia. In contrast, the absence of the mucosal wave and a restricted or absent vibratory amplitude indicates invasion into the deeper vocal fold structures [10].

Embryonic stem cells rely on the SOX2 gene to remain pluripotent. In other words, it allows a single cell to undergo cell differentiation into many cell types. Embryonic stem cells cannot differentiate, self-renew, or have their cell destiny decided without SOX2. It is unclear what function the SOX2 gene has in head and neck squamous cell carcinoma, particularly in laryngeal instances, although it is linked to the formation of malignant tumors such as lung tumors, glioblastoma, and some forms of SCC [11].

We acknowledge that in current clinical practice, many laryngologists treat selected vocal fold leukoplakia lesions with laser excision without obtaining a biopsy, and therefore would not routinely request molecular analyses such as SOX2 expression. Our study does not advocate for the immediate clinical use of SOX2 testing; rather, it provides preliminary mechanistic evidence that SOX2 may be involved in early steps of laryngeal squamous cell carcinoma (LSCC) development. If these findings are validated in larger, prospective cohorts, a better understanding of SOX2’s role could eventually help refine treatment decisions. For instance, by identifying patients with low-grade lesions who harbor molecular features associated with a higher risk of progression, thereby supporting a more proactive approach (e.g., early laser intervention) rather than surveillance alone. Conversely, absence of elevated SOX2 might reinforce the safety of a watchful-waiting strategy. Thus, the primary contribution of this work lies in its mechanistic exploration of SOX2 within the neoplastic spectrum of vocal fold lesions, offering a foundation for future studies aimed at translating molecular markers into clinically actionable algorithms.

The clinical and morphological examinations showed no significant differences among the histopathological groups in this study.

Rzepakowska et al. [6]found that not only the irregular texture but also the heterogeneous color and the marked thickness significantly correlated with malignancy. Furthermore, Research by Chen et al. established a significant link between the physical appearance of vocal fold leukoplakia and its underlying pathology. Their findings indicated that flat, smooth lesions were generally not dysplastic. In contrast, lesions that were smooth yet elevated frequently corresponded to mild or moderate dysplasia. The most severe outcomes (severe dysplasia and carcinoma) were predominantly observed in lesions with a rough surface texture [12]. Zhang et al. [5] found that lesions with a bulging and rough appearance showed a significant association with either malignant transformation or severe dysplasia. In contrast, lesions that were flat and smooth correlated with pathology findings of only mild or no dysplasia.

The discrepancy between our results and the previously mentioned studies was likely due to substantial methodological differences. Our study was restricted by factors including small, fragmented biopsies (which were necessitated by the requirement for further genetic analysis) and subjective visual evaluations. In contrast, other studies typically enjoyed advantages from larger tissue samples [5]. They encompassed various pathologies spanning from only leukoplakia [6] to verrucous and exophytic lesions with erosion or ulceration [5] (which skewed the results). Additionally, their studies were conducted retrospectively, employing older, different laryngo-endoscopic technologies [5, 10], and categorized the lesions into other distinct histopathological subgroups [5, 10, 13].

In our study, the stroboscopic examination was compared with the histopathological results. A significant difference existed between the laryngo-stroboscopic parameters (mucosal wave, amplitude of VF vibration, non-vibrating segment, and symmetry in phase) and the different histopathological groups. Patients who were suspected of having low-risk lesions (benign or low-grade dysplasia) were suggested to have a normal mucosal wave; patients with high-risk lesions (high-grade dysplasia, carcinoma in situ or SCC) to have a decline or absence.

The results we obtained were consistent with Rzepakowska et al. [6] reported that reduced or absent mucosal waves in VF leukoplakia had the strongest correlation with malignancy. This is consistently supported by the broader literature, where the collective findings of El-Demerdash et al. [10], Leduchowska et al. [13], Cui et al. [14], Li et al. [15], and Krausert et al. [16]confirm that stroboscopic assessment of mucosal wave abnormalities serves as a critical non-invasive predictor for both high-grade dysplasia and invasive carcinoma, as these wave patterns are directly dependent on the structural integrity of the vocal fold lamina propria.

In a divergent study, Colden et al. [17]evaluated the efficacy of stroboscopy in predicting the depth of invasion in keratotic lesions. Frequent occurrences of impaired mucosal wave and amplitude were noted in non-invasive precancerous lesions, which were attributed to reduced epithelial pliability caused by bulky keratosis and underlying changes such as fibrosis. Consequently, they argued that these stroboscopic parameters were unreliable indicators for differentiating cancer from atypia or for assessing the depth of malignant invasion.

The SOX2 gene expression was measured using real-time PCR in this study. Compared to the low-risk group (benign and low-grade dysplasia lesions), the expression was greater in the high-risk group (high-grade dysplasia lesions, cancer in situ and SCC). Also, there was a moderate positive correlation between Log SOX2 expression and the histopathological findings, according to the correlation analysis (*r* = 0.514, *P* < 0.001).

Currently, many laryngologists treat vocal fold leukoplakia with laser excision without biopsies, so SOX2 testing is not routine. This study offers preliminary evidence that SOX2 may be involved in early laryngeal cancer development, not yet advocating for immediate clinical use. If validated in larger studies, understanding SOX2 could help tailor treatments by identifying high-risk lesions for earlier intervention or confirming when surveillance is safe.

A strong correlation wasn’t achieved. That could be explained primarily by the significant heterogeneity of leukoplakic patches. This means that a single biopsy may not capture all molecular hotspots, as the sample is divided into two parts for histopathological examination and genetic study. As a result, a focal area of high SOX2 gene expression (a hotspot) may be missed, creating a sampling bias that obscures true correlations [18]. The functional impact of SOX2 was also heavily influenced by specific cohort factors, such as smoking status or genetic background, as well as the technical aspects of gene detection [19].

Additionally, the inherent complex biology of SOX2 may potentially act as an initial trigger in early dysplasia rather than a continuous driving force, resulting in a biphasic expression pattern that complicates a straightforward linear relationship [20, 21]. During early carcinogenesis, SOX2 is upregulated to enhance cellular survival and confer stem-like properties in precancerous lesions facing genomic instability [22]. In contrast, its expression is frequently suppressed in advanced malignancies through epigenetic mechanisms and pressures within the tumor microenvironment [23]. This subsequent downregulation promotes a phenotypic switch from pluripotency to processes that drive invasion and metastasis [24].

Our data also revealed that multivariate binary logistic regression analysis revealed Log SOX2 gene expression as an independent predictive marker for progression from low-risk lesions to high-risk lesions (*P* < 0.001).

In line with our results, Diaz et al. [7] examined 94 individuals who had vocal fold precancerous tumors. Using immunohistochemistry, they looked for signs of SOX2 protein expression in the samples. In addition, 55 of the samples were tested for SOX2 gene amplification with real-time PCR. The results showed that 18 cases had positive SOX2 gene amplification when the SOX2 gene expression was greater than 1.75. Additionally, they discovered that gene expression correlated with lesion severity; specifically, SOX2 gene expression was positive in 17 out of 49 instances of high-grade dysplasia and 1 out of 6 cases of low-grade dysplasia. It was not statistically significant that one group was different from the other. In instances of malignant transformation into SCC, there was a significant correlation between SOX2 gene expression (by PCR) and SOX2 protein (by immunohistochemistry). Furthermore, they asserted that in patients with precancerous lesions, SOX2 expression significantly predicted the development of laryngeal malignancy.

Consistent with the oncogenic role of SOX2, studies by Zhang et al. [21] and Schröck et al. [25] confirm that its overexpression drives tumor proliferation, stemness, and metastasis [21], correlating significantly with poor patient prognosis and chemoresistance in HNSCC [25], thereby underscoring its potential as a therapeutic target.

In contrast to our results, Chung et al. [11]analyzed SOX2 gene expression in a cohort study including head and neck SCC. They assessed the effect of radiation on SOX2 gene expression by grouping patients into those with high and low levels of the gene. A better outcome was seen in the SOX2 high subgroup. Their reasoning was that this was due to SOX2’s ability to guard against cancer by increasing the rate at which cancer cells die. Possible explanations for the contradictory findings include tumor-specific genetic abnormalities, hypoxia, cell cycle, apoptosis, growth hormones, and radiosensitivity.

A recent meta-analysis further found that in non-small-cell lung cancer, SOX2 expression was linked to a better prognosis [26].

Züllig et al. [27] discovered that poor survival was linked to low SOX2 expression, whereas improved prognosis was linked to high SOX2 levels, which were associated with fewer lymph node metastases. Bayo et al. [28]shown that SOX2 inhibits the migration of tumor cells in Squamous cell carcinoma of the head and neck (SCC) cells, and that a lack of SOX2 expression might be used to identify individuals at high risk of treatment failure. Furthermore, in cervical malignancies of unknown origin and head and neck SCC, poor outcomes have been associated with decreased SOX2 expression of genes on chromosome 3q26.

## Conclusions

The integrated application of laryngostroboscopy and histopathological evaluation provides valuable diagnostic insight into the vocal fold irregular lesions across the neoplastic spectrum. Elevated SOX2 expression was observed in high-risk lesions, and multivariate analysis suggested it has a probable role in malignant transformation. These findings underscore the need for continued molecular research to elucidate the mechanisms of early laryngeal carcinogenesis and to identify robust biomarkers for early detection and risk stratification. This exploratory study provides a foundation for larger-scale investigations into the molecular pathways of laryngeal carcinogenesis.

## Limitations

This study has several limitations that should be considered. First, its single-center design and relatively small sample size may limit the generalizability of the findings. The absence of normal control tissue prevents the establishment of a definitive baseline for SOX2 expression in healthy vocal fold epithelium. Furthermore, the analysis was confined to vocal fold lesions, without comparison to similar pathologies in other anatomical sites, narrowing its contextual relevance. A significant interpretive challenge arises from the dual and contrasting roles reported for SOX2, which can exhibit both carcinogenic and tumor-suppressive functions, leaving its precise mechanistic role in this context unclear. Finally, the assessment of SOX2 expression at a single time point, without longitudinal follow-up, precludes any analysis of its dynamic changes over the course of disease or treatment.

## Data Availability

Data is available on reasonable requests from the corresponding author.
